# Varicella associated pneumoniae in a pediatric population

**DOI:** 10.1186/s13052-017-0366-8

**Published:** 2017-05-30

**Authors:** Elena Bozzola, Guido Castelli Gattinara, Mauro Bozzola, Nadia Mirante, Marco Masci, Chiara Rossetti, Andrzej Krzystofiak, Luciana Nicolosi, Renato Cutrera, Laura Lancella, Alberto Eugenio Tozzi, Alberto Villani

**Affiliations:** 1 University/hospital Department of Pediatrics, Pediatric and Infectious Diseases Unit, Bambino Gesù Children’s Hospital, IRCCS, Rome, Italy; 2 Internal Medicine and Therapeutics Department, Pediatrics and Adolescentology Unit, University of Pavia, Fondazione IRCCS San Matteo, Pavia, Italy; 3 University/hospital Department of Pediatrics, Pulmonology Unit, Bambino Gesù Children’s Hospital, IRCCS, Rome, Italy; 4 Sanitary Direction, Bambino Gesù Children’s Hospital, IRCCS, Rome, Italy; 5 IRCCS Bambino Gesù Children Hospital, Rome, Italy

**Keywords:** Varicella, children, pneumoniae complication, hospitalization, vaccine

## Abstract

**Background:**

Varicella pneumonia has been studied extensively in adults; it may also affect children and may require hospitalization.

**Methods:**

We examined pneumonia complications in children hospitalized for varicella, over a 13 year period.

**Results:**

Pneumonia occurred in 8.2% of children hospitalized for varicella. The median length of hospitalization was 6 days. No statistically significant difference in length of stay was detected between immunodepressed children and previously healthy children. The hospitalization was on average shorter in patients who started antiviral therapy within 24 h of varicella onset. None of the included patients had been previously immunized for varicella.

**Conclusions:**

Our results support the need for increased awareness of current varicella prevention recommendations among both immunocompetent and immunodepressed individuals. In children affected by varicella, prompt antiviral therapy may be indicated to reduce the number of days of hospitalization.

## Background

Varicella is an exanthematous infectious disease that usually affects children. It generally has a benign course, but in some cases it may require hospitalization [1, 2]. Among complications, varicella may cause pneumoniae [3].

## Aim of the study

The aim of the study was to investigate the incidence of pneumoniae complications among children hospitalized for varicella at the Bambino Gesù Children’s Hospital, Roma, Italy.

## Methods

We retrospectively included patients younger than 18 years old admitted to the Bambino Gesù Children’s Hospital for varicella over a 13-year period (from January 2003 to January 2016). The parents of all the subjects gave their informed consent for inclusion in the study. The study was conducted in accordance with the Declaration of Helsinki. In all patients, varicella was clinically diagnosed, according to the literature. Molecular diagnosis of chickenpox by polymerase chain reaction is required only in case of doubt. 3) Chest X-rays were performed at hospital admission and were evaluated by a pediatric radiologist. In the literature, a varicella-related complication is a condition occurring within 14 days of onset of varicella that may have been influenced by varicella-zoster virus infection [4]. In accordance, pneumoniae was diagnosed as a complication of varicella when an interstitial infiltration or nodular lesion was presented as a radiological finding within 2 weeks of the appearance of the varicella rash [5]. Microbiological blood analysis (culture, viral PCR), and analysis of nasopharyngeal aspirates and sputum were performed in order to detect viral or bacterial co-infections, depending on medical prescription. The patient’s state of health was determined according to two categories: previously healthy if no chronic health problems were reported by the family in the interview; immunosuppressed in one of the following circumstances: currently receiving chemotherapy or radiotherapy, immunosuppression treatment for transplantation, intravenous steroid therapy, congenital immune deficiency, or HIV infection regardless of the level of immunodeficiency [6].

### Statistical analysis

The continuous variables are reported as median values (range) because the variables did not present a normal distribution, and categorical data are given as number of cases and percentages. The Mann-Whitney U test was performed to compare the two groups (health vs immunodepressed children). A *p* value <0.05 is considered as significant. Data were analyzed using STATA for Window release 14.

## Results

Among all the children hospitalized in the study period, 825 were admitted for varicella. The age of patients was on average 6.6 years (range from 2 weeks to 17 years and 6 months). Out of 825 patients, 68 were diagnosed with pneumoniae within 2 weeks of varicella onset (8.2%).

At hospital admission, children affected by pneumonia presented respiratory symptoms. Most of them referred with cough (60 patients; 88.2%), fever higher than 38.5 °C (40 patients; 58.8%), tachypnea (20 patients; 29.4%), dyspnea (15 patients; 22%) and chest pain (6 patients; 8.8%). At physical examination, crackles were reported in 62 patients (91%), decreased breath sounds in 48 patients (70.5%) and dullness at percussion in 5 patients (7.3%).

**Fig. 1:**
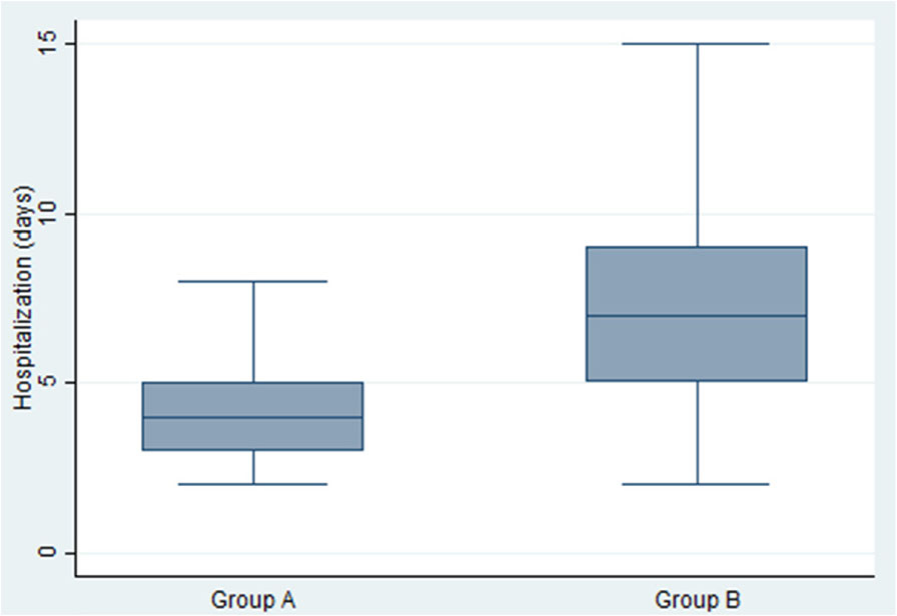
Length of Hospitalization (days) in patients with antiviral therapy started <24 h from the onset of varicella (group A – 13 Patients) and in patients with antiviral therapy started >24 h from the onset of disease or without antiviral therapy (group B – 41 Patients)

Among patients affected by pneumoniae, ten (14.7%) had a pre-existing health problem. In detail, two of them had a malignancy, six a cardiopathy, one cystic fibrosis and one was receiving immunosuppressive therapy for a liver transplantation. The median age of patients with varicella pneumonia was 3 years and 4 months (range from 2 months to 10 years). Ten of the patients were below the age of 1 year. The median time from varicella onset to hospitalization was 4 days (range from 0 to 14 days). The median length of hospitalization was 6 days (range from 2 to 67 days). No statistically significant difference in length of stay was detected between immunodepressed children (8 days) and previously health children (6 days). Hospitalization was on average longer in patients with concomitant pleural effusion (13 days) than in those with more than one pneumoniae obliteration (12 days) and those with a single localization (7 days). One patient affected by leukemia died during the hospital stay. Two patients were admitted to pediatric intensive care and eight received oxygen supplementation during hospitalization. Four previously healthy patients underwent surgery for massive pleural infusions. In these cases, the median length of stay was 29 days (range from 16 to 55 days). All the patients included in the study underwent nasopharyngeal aspirate testing. An adenovirus co-infection was detected in one case. Ten patients, instead, were asked for sputum: Streptococcus Pyogenes was found in four cases, Haemophilus Influenzae in two cases and Nocardia Transvalentis in one case. Forty patients underwent microbiological blood examination in order to detect viral or bacterial superinfections. An Epstein Barr Virus co-infection was detected in three cases by PCR. The blood culture for aerobic and anaerobic bacteria was negative in all patients. Fourteen patients presented more than one varicella complication apart from pneumoniae. In detail, five of them were diagnosed with cellulitis, one with cerebellitis, one with encephalitis, two with diarrhea, four with cutaneous superinfections and one with arthritis. In these patients, the hospitalization was longer (9 days) than in those with just pneumoniae complication (6 days) (*p* = 0.05). Among patients with just pneumoniae complication, 13 started antiviral therapy with acyclovir at home within 24 h of the exanthema onset. Table 1 summarizes the clinical characteristics of the patients. Patients who promptly started antiviral therapy had a statistically significant shorter hospitalization than the others (4 vs 7 days; *p* = 0.001). The decision of whether to start antiviral therapy depended on the medical doctor’s advice (Fig. 1).

**Table 1:** Clinical characteristics of the patients with antiviral therapy started <24 h from the onset of varicella (group A) and of patients with antiviral therapy started >24 h from the onset of disease or without antiviral therapy (group B)

	Group A	Group B
Number of patients	13	41
Age median (range)	3.85 years (6 months-10 years)	3.56 years (2 weeks-9.5 years)
Sex (Male%/Female%)	53.8%/46.2%	51.2%/48.8%
Number of patients with a pre-existing health problem (%)	15.3%	12.2%

Children who promptly received therapy had the same median age as the others (3 years and 9 months vs 3 years and 3 months; *p* = 0.09). None of the children included in the study had been previously vaccinated for varicella.

## Discussion

In this study we analyzed varicella-associated pneumoniae in hospitalized children over a 13-year period. Few reports focusing on pneumoniae complications of varicella in pediatric age are available in the literature as pneumonia is frequently detected as an adult varicella complication [7, 8]. Previous studies on childhood varicella complications are in line with our results, as they reported an incidence of varicella pneumoniae variable from 5.61% to 30.3% among children hospitalized for varicella [5, 9, 10]. The outcome of varicella is thought to depend upon the severity of the disease and the immune state of the patient. Varicella complications are reported to be rarer in immunocompetent children than in immunodepressed ones [4, 5]. Some authors claim that varicella is especially severe in immunocompromised hosts, for whom there is an increased risk that the virus will disseminate throughout their organs [3, 11]. Other studies demonstrated that most patients with varicella complications had no severe underlying immunocompromising conditions [12, 13].

In a previous study, comparing healthy and immunocompromised children, pneumonitis was found to be the most common complication of varicella infection in previously ill patients [5].

In our study, on the contrary, pneumonia associated with varicella infection mainly affected previously healthy children (85.3%). The mean length stay was similar in both immunocompetent and immunodepressed children. Nevertheless, we have to underline that the only patient who died during the study period was an immunocompromised host due to a malignancy. Antiviral therapy may also affect the prognosis when prescribed within the first 24 h after the rash develops in both immunocompetent and immunodepressed hosts [3, 14–16]. In our study, we found that the length of hospitalization was shorter in those who started the therapy promptly. In Italy, in unvaccinated subjects, oral antiviral therapy is currently recommended for immunocompetent individuals without evidence of complications and for immunocompetent children and adolescents who are at increased risk of developing complications [17].

According to international indications, medical doctors are more likely to prescribe antiviral therapy to children with a pre-existing medical condition as they are aware of the likely complications. However, some pediatricians may prescribe antivirals also in healthy children, in order to avoid complications.

A risk factor for longer hospitalization in patients affected by varicella associated pneumoniae is having any other complication at the same time.

Finally, another important finding is that no children had received immunization in the past, even though 58 patients were older than 1 year and consequently potentially vaccinable.

## Conclusions

We have reported our experience as we consider that data on varicella complications are useful to improve local immunization strategies. In fact, varicella affects not only children but also represents a medical burden for society. The median cost for a two-day hospitalization of a child affected by a complicated varicella is high (about 1660 euro) [18].

Our results support the need for increased awareness of current varicella prevention recommendations among both immunocompetent and immunodepressed individuals, which includes the targeting of susceptible household contacts for vaccination.

In our study, we point out that pneumonia associated with varicella may require hospitalization also in children without pre-existing illness. Prompt antiviral therapy may be indicated to reduce the number of days of hospitalization in affected children.

Further studies with larger numbers of patients may be useful to support our conclusions.
